# The Value of Tracking Data on the Behavior of Patients Who Have Undergone Bariatric Surgery: Explorative Study

**DOI:** 10.2196/27389

**Published:** 2022-05-06

**Authors:** Dirk Versteegden, Magaly van Himbeeck, Anne W Burghoorn, Peter Lovei, Eva Deckers, Jos-Marien Jansen, Simon Nienhuijs

**Affiliations:** 1 Department of Surgery Catharina Hospital Eindhoven Netherlands; 2 Philips Experience Design Eindhoven Netherlands; 3 Department of Industrial Design Eindhoven University of Technology Eindhoven Netherlands

**Keywords:** home telecare, bariatric surgery, data-enabled approach, mobile phone, smartphone, mHealth, mobile health, data tracking, tracker, app

## Abstract

**Background:**

To maintain the benefits of a bariatric procedure, patients have to change their lifestyle permanently. This happens within a context of coresponsibilities of health care professionals and their social support system. However, most interventions are focused on the patient as an individual. In this explorative pilot study, behavioral, contextual, and experiential data were gathered to obtain insight on coresponsibility.

**Objective:**

The aim of this study is to explore the use of trackers by patients who have undergone bariatric surgery in a data-enabled design approach.

**Methods:**

Behavioral and contextual data on the households of patients who have undergone bariatric surgery were explored using a smartphone with an interactive user interface (UI), weight scale, activity bracelet, smart socket, accelerometer motion sensor, and event button to find examples of opportunities for future interventions.

**Results:**

A total of 6 households were monitored. Approximately 483,000 data points were collected, and the participants engaged in 1483 conversations with the system. Examples were found using different combinations of data types, which provided the obesity team a better understanding of patient behaviors and their support system, such as a referral to a family coach instead of a dietician. Another finding regarding the partners was, for example, that the conversational UI system facilitated discussion about the support structure by asking for awareness.

**Conclusions:**

An intelligent system using a combination of quantitative data gathered by data tracking products in the home environment and qualitative data gathered by app-enhanced short conversations, as well as face-to-face interviews, is useful for an improved understanding of coresponsibilities in the households of patients who have undergone bariatric surgery. The examples found in this explorative study so far encourage research in this field.

## Introduction

**Figure 1:**
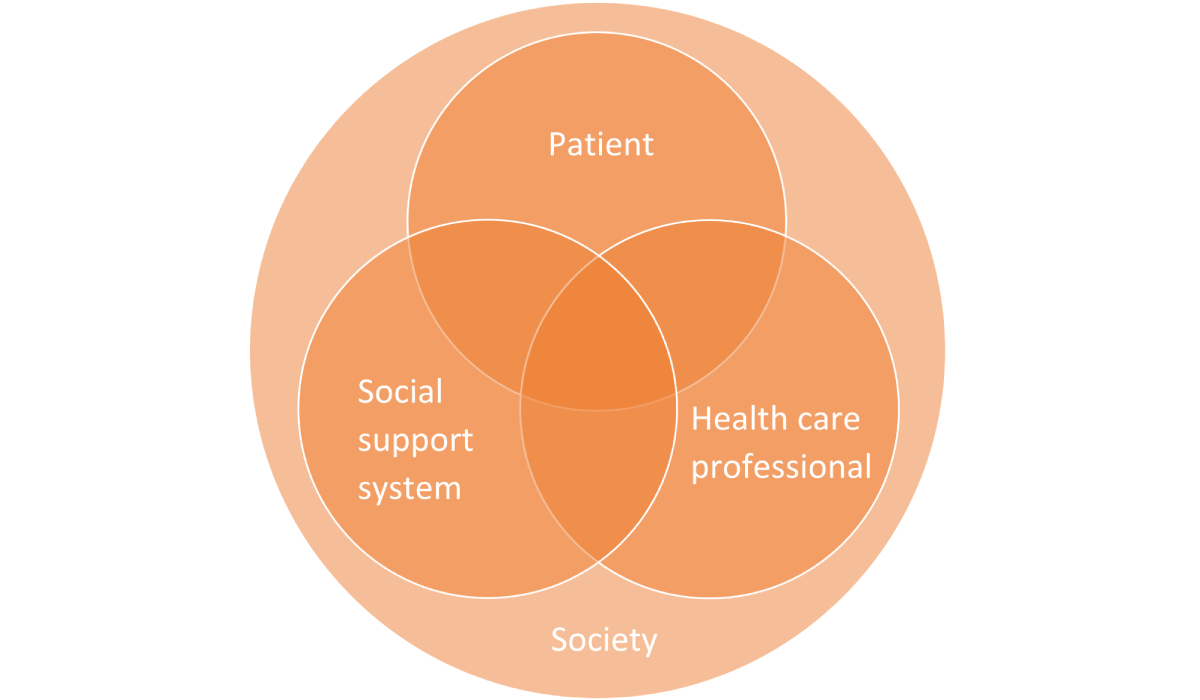
Coresponsibility system.

Overweight and obesity are steadily growing into one of the largest threats to human health in this century, as almost 2 in 5 people are overweight worldwide [1]. Bariatric or weight loss surgery has been used to treat patients with morbid obesity for decades with a proven long-term effect [2]. Worldwide, more than 800,000 procedures are performed annually [3]. Key aspects of a successful treatment include multidisciplinary preoperative screening and postoperative guidance [4-6]. Even though most aftercare programs are focused on the importance of a long follow-up by a dedicated obesity team, and there is increasing knowledge on primary care for long life guidance thereafter, the time and resources used could not be enough for individual patients [7]. Obesity teams gather as much information as possible from the interviews, anamnesis, and group sessions. However, this is time-consuming, subjective, and probably incomplete. Besides lab results for medical follow-ups, other frequently used aids are questionnaires, diet diaries, and exercise tests. As obesity is undoubtedly multifactorial in origin, more sources of data can be useful, for instance, the frequency and duration of exercises and meals. Such information can be useful for the patient and for the obesity team as both have their part in the responsibility to maintain the benefits of a bariatric procedure. Frequently, behavior is influenced by those living with the patient. In most cases this will be a partner, but it could also be parents, children, housemates, close friends, or even colleagues. This could pose a challenge to successfully change behavior postoperatively, as preoperative behavior is often intertwined with a person’s social life. As this behavior is crucial for a successful bariatric intervention, the social support system has a share in this responsibility as well. Connecting these responsibilities within limited time and with limited information is demanding. An answer to this demand could be tracking data from households, using several devices with information originating from the patients, as well as others in the direct social support system. A visualization of these coresponsibilities is suggested in Figure 1 [8,9]. Creating an interactive system with more data for these parties could enhance this coresponsibility and, therefore, improve weight loss and quality of life for a long period. In this explorative study, such an interactive system was created. The aim of this study was to assess the value of an interactive system with tracking data from a household as a social support system for the patients who had undergone bariatric surgery.

## Methods

### Ethics Approval

This pilot study was part of a clinical trial entitled “Together in Shape.” Its setup as a therapeutic intervention study was approved by the Philips Internal Committee of Biomedical Experiments, the medical ethical review board of Medical Research Ethics Committees United (MEC-U) and Central Committee on Research Involving Human Subjects (NL63252/100.17), and the local feasibility committee (CZE-2018.06).

### Setting and Selection Criteria

The setting was the obesity center of Catharina Hospital where up to 1000 patients are treated annually. Normally, a patient has 27 contacts individually or in group with the multidisciplinary team during 5 years of follow-up. Additional visits or telephone-based consultations are provided whenever necessary or requested. Only by exception is the guidance program transferred to primary care [10]. Information about the study was provided through the center’s eHealth portal. Selection criteria were patients in their postoperative trajectory after bariatric surgery, who had at least 1 person living nearby, could speak the Dutch language, were above the age of 18 years, willing to participate and to have house visits by the research team, and who signed an informed consent form. Eligible patients were informed by the research team, and in-depth information about the study was provided. A series of consecutive patients who had undergone bariatric surgery were informed about the study until 6 households were included. The number of inclusions was limited due to the explorative design of the study. Each household was followed for approximately 5 to 6 weeks.

#### Study Design

A data-enabled design approach was used, which means that the collected contextual data were used during the process to continuously enhance, update, and shape the design [11]. Such a study usually consists of 2 phases: the *contextual phase*, to get a good conception of the setting; and the *informed phase*, when design interventions are tested out in the participants’ own context—in this study, their home. In the *contextual* *phase*, the focus was on collecting and determining which data was interesting and how to display this comprehensibly. This was mainly done by conversations with the participants, the research team, and health care professionals. No interventions were done in this phase. This was introduced in the *informed phase*, such as coaching and giving tips.

Semistructured interviews were used to gain insight on the lifestyle, daily routine, home environment, and social system of the patients. The interviews were planned beforehand and took about 2 hours; they were based on a predefined questionnaire that was compiled before the start of the study and was approved by the MEC-U. The interviews were important as they were used to correlate data points to behavior and put them into a contextual setting. During these interviews, several themes were discussed and clarified such as the general condition of the subjects, daily routine, mental health, commitment, satisfaction, possible changes to the previously mentioned elements due to the trackers, utilization of trackers, technical problems with the trackers, and unexpected outcomes. The interviews allowed for time to have practical discussions about the interactive system, the app, and to gain insights on the lifestyle, routines, and social system of the patients. This could further inform the research team of the trackers that could be used. Potential changes in the data tracking and system setups were part of this process. A translated transcription of the semistructured interviews is provided in Multimedia Appendix 1. The interviews were transcribed and coded for analysis. Furthermore, nonstructured interval interviews were held on demand during the follow-up period to gain contextual insights into the gathered data points.

For this study, the 3 pillars of the coresponsibility system (Figure 1) that were analyzed were the obesity team (as health care professionals), the patient, and their partner (as the social support system). It was recognized by the research team that a partner is only a part of a social support system. It can also include and is not limited to other individuals such as housemates, children, parents, a close friend, colleagues, etc. In this study, the role of the partner in the social support system was the subject of the investigation to simplify the methods and results. An interactive system was created that gathered data from different sources such as medical records, situated contextual data (eg, physical activity, mental health, and nutrition), and self-reported data (eg, which family member is cooking), and consisted of different communication platforms for each of the stakeholders to facilitate the presentation and sharing of personalized coaching content for the patients who had undergone bariatric surgery and their partners. The specific collected data types and the specific content of the communication functionalities were not preset and were subject to change during the study period. Therefore, there remained a possibility to experiment with different data types and features to explore multiple designs for benefitting coresponsibility based on care questions and priorities.

**Figure 2:**
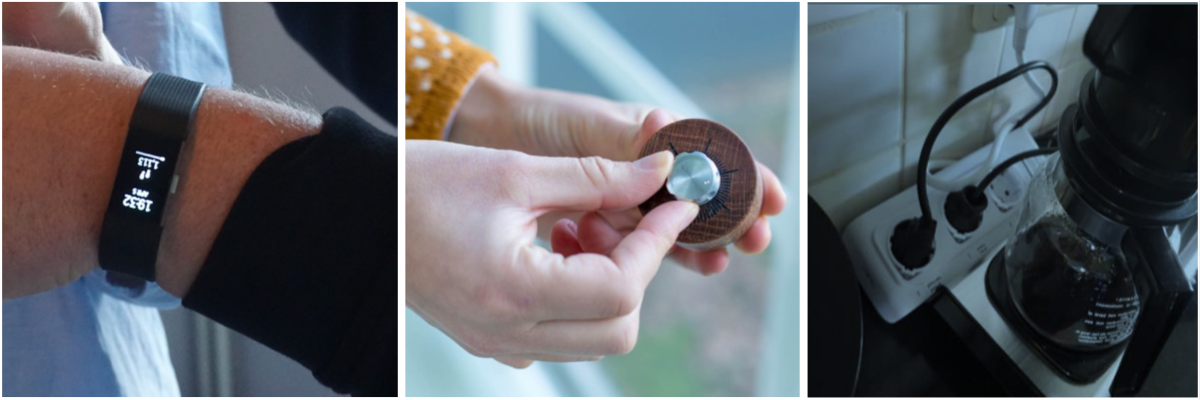
Three examples of trackers used to collect contextual and behavioral data: activity bracelet (left), event button (middle), and smart socket (right).

The intelligent system consisted of 4 elements: data trackers, a mobile phone app, an obesity team dashboard, and a research dashboard. Three types of data trackers were used (Figure 2), which are as follows: *personal data*, *contextual data*, and *open data*. *Personal data* (physical activity bracelet and weight scale) was used for measuring number of steps, heart rate, and weight. These devices were used to give patients more insights on their weight loss progress and physical activity; the activity bracelet could interact with the mobile phone app and the dashboards. *Contextual data* (smart sockets and accelerometer) was used for measuring the use of devices (eg, television, household equipment) and movement (eg, opening cabinets or picking up sports equipment); these were used to track activities in home environment. *Open data* (smart buttons and rotary knobs) was used for measuring events (eg, cooking) and experiences (eg, how bored I am).

**Figure 3:**
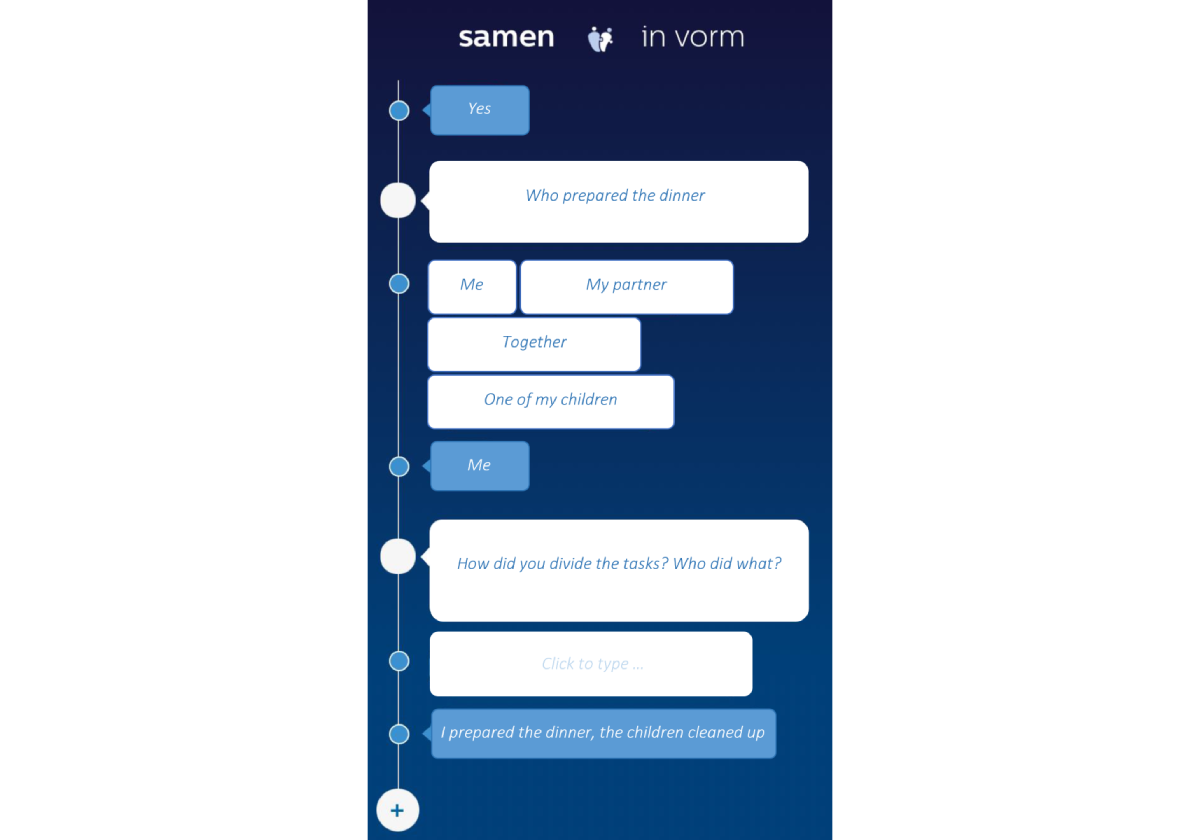
Screenshot of the mobile app.

Patients and their partners were able to use a phone with a study app conversational user interface (UI) to participate in preset conversations based on data triggers (eg, when the accelerometer registered motion in the kitchen cabinet) with preset times, or when they were initiated by the research team. An example is shown in Figure 3. Patient and partner each had their own phone and app and received individual conversations. They were instructed about the trackers and the apps and how to use them properly. The trackers were placed on places and objects in their house after agreement with the subjects.

**Figure 4:**
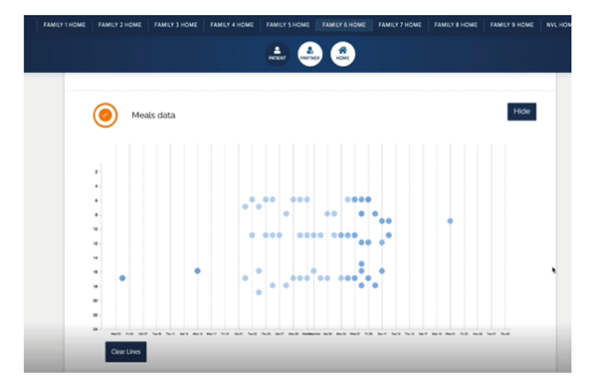
Example of data on meals.

A large number of data points were expected to be collected. The activity bracelet gathered daily physical activity summary and sleep data. The weight scale generated data points upon weight measurement. The motion sensor produced data points when a movement was registered. App analytics were recorded when patients sent or received messages or when the app was opened, interacted on, and closed. The smart buttons and the rotary knobs recorded data points when patients interacted with the buttons and based on the value they entered. To process these data points, a few steps were taken. The data were preprocessed by filtering out test data, removing outlines, and removing data points that were sent over by the devices whether or not they were working (eg, battery status). Next, script-based data analysis was performed based on clinical guidelines (eg, number of recommended steps, daily recommended meals). Lastly, dashboards were designed to visually interpret the data. An obesity team dashboard was produced to get insights on the data, and more importantly, to discuss within the research team which data were valuable. An example of the dashboard is shown in Figure 4. This visualization shows time points when a subject registered a meal. Because the types of data were adaptable and decided in negotiation with the participants, the available data were different per household. Likewise, a dashboard was built for research purposes and used by the researchers to setup, modify, and analyze the study as well as trigger the personalized coaching content in the chatbot communication system.

## Results

In total, 12 participants were included in the study: 6 patients and their partners (Table 1).

The data trackers and app analytics generated around 483,000 data points, and the participants engaged in 1483 interactions with the system. The mean number of daily app interactions ranged from 7.2 to 16.9. This information was transferred into a data visualization timeline, where the interventions (eg, providing suggestions for an exercise or a recipe) were also taken into account. The data points are the sum of the collected personal, contextual, and open data points described in the previous section. The most contextual data points were retrieved from the connected power sockets that were attached to the participants’ televisions and kitchen appliances. However, the large amount of data points was mainly due to power usages and not turn on or turn off events. The second most retrieved contextual data points were from the motion sensors. The participants themselves reported 641 events using the open data trackers (eg, smart buttons indicating an event or rotary buttons for emotion assessment). Lastly, the personal data points included the daily summaries of the activity tracker’s sleep and activity data.

**Table 1:** Study population characteristics.

Household^a^			Study phase		Postoperative time		Number of daily app interactions, mean (SD)
1	Anna and Alex	Contextual		10 months		16.9 (37.7)	
2	Bella and Brian	Contextual		12 months		7.2 (6.7)	
3	Chloe and Chris	Informed		Direct (first operative week)		10.6 (14.5)	
4	Diana and David	Informed		4 months		11.1 (7.7)	
5	Emily and Eric	Informed		5 months		9.8 (7.6)	
6	Fiona and Felix	Informed		12 months		10.1 (4.4)	

^a^Fictive names were used.

Findings that focused on coresponsibility between the patient and the obesity team were analyzed, and opportunities for interventions were consequently identified. Examples of these findings, which led to insights or changes in the intelligent system, will be explored below.

One example describes an interesting finding about household dynamics around food choices. In this household, the deep fryer was used more often than originally indicated in the meeting with the patient and the partner before the system was placed in their home. The household indicated that they eat fries once a week. However, the smart socket showed usage of the deep fryer 6 out of 7 days. After this was brought up to the household, it became clear that this was due to a disagreement on what to eat between the patient, the partner, and their children. Instead, they chose to eat fries to accommodate everyone’s eating desires.

The system permitted assessment of the effectiveness of an intervention, illustrated by an example on physical activity where feedback in the app led to changes in the intervention. In the same household of example 1, the frequency and duration of exercise was adequate, although weight loss was still unsatisfactory. In an effort to explain this, the physiotherapist had to look for other reasons for this insufficient weight loss. The patient noticed that by using the app after scoring the intensity of exercises for a week, she might need to increase the intensity rather than the duration or frequency to improve weight loss.

In another household, an example of coresponsibility between the obesity team and the patient was found. The participant’s health record indicated low self-esteem. Her study data showed that she had a very good workout regime. She was advised to complement her workouts with the conversation app, which she found very difficult to do. Her realization supported the willingness for additional psychological help.

Two other examples were on the coresponsibilities between the patient and their partner. Involving the partner more in the care trajectory could be beneficial but difficult to organize. Therefore, the conversational UI system facilitated the discussion about the support structure by asking for awareness of the amount and type of support that the patients received from their partners. In one case, the partner was impressed that the patient was capable of reaching results on her own, whereas the patient still wanted to have support. Receiving questions on the app about the partner’s support triggered a discussion between the patient and their partner about this subject. In another case, the patient realized that she had asked for support and received it but had found it difficult to appreciate it. In the face-to-face conference afterwards, the couple pointed out that the system had made them both feel as if they were part of the same lifestyle rather than having two separate lifestyles.

Upon asking, all the partners commented that they wanted to support the patients; however, they also felt that altering their own behavior was not part of that. One example of how the intelligent system could influence the behavior and coresponsibility of the partner was found in household 6. The partner received a coaching message on the app to surprise the patient with a healthy alternative. He agreed, got positive feedback, and asked for more recipes later on.

## Discussion

### Principal Results

The behaviors and experiences of patients who have undergone bariatric surgery in relation to their lifestyle change take place within a social context and interaction with others. Such a context is quite complex to investigate and, therefore, influence. On the other hand, it is important for a patient’s long-term outcome as well as for their households. One possible approach toward this is to get insights into behaviors in the home environment using a data tracking system. Data tracking and interviews might provide an understanding of coresponsibilities in these households. By using this approach, this explorative study revealed some examples of intermingling responsibilities of the household members (sometimes the children but mostly the partners), members of the obesity team, and the patients. A limitation of this study was that the results were case-specific and, therefore, not reproducible. They merely underlined the substantial variety in social dynamics in these households. A combination of different household members, each having different values and interests, was linked to a healthier way of living. In order to help the patients’ household members, by design, to be supportive toward a healthy lifestyle change, they need to be approached in a way that fits these values and interests and the roles they take or could possibly take.

These data were regarded as a useful addition to the information normally gathered in short consultations and through some body measurements. For instance, the trackers showed that the intensity of the exercise, rather than frequency and duration, increased effectiveness. Another example showed that patients might, intentionally or unintentionally, underreport unhealthy intake, and an intelligent tracker system could reveal this. Having access to this information during a postoperative checkup might lead to choosing other intervention options. For instance, instead of a referral to a dietician, a family therapist might yield better results in some cases.

The relationship between the physicians’ and patients’ coresponsibilities is in some cases unilateral: a physician is restricted to advise on treatments and lifestyle changes based on what a patient communicates in a consultation and with a few measurements (eg, BMI, comorbidity status). It is up to the patient what to do with this advice. By using an intelligent data tracking solution, physicians could be able to control or evaluate a therapy. A recent study also showed that high compliance can be achieved by the use of supervised home monitoring [12].

The examples found in this study show opportunities for designing new intelligent systems. However, privacy concerns might limit the effectiveness of this approach. Therefore, for future research, we emphasize the importance of giving control of data sharing to the users themselves. Findings suggest that the effect of the partner is substantial. This would be an interesting field for future work, as the role of the partner in adjusting lifestyle remains unclear. Further research is also needed to discover to what extent the role of others (eg, children, friends, colleagues) is important in maintaining a healthy lifestyle postoperatively.

Another concern might be compliance. Prior studies have presented conflicting results in terms of compliance and reach of telehealth solutions to the use of telemonitoring intervention, ranging from 50% up to 94%. These studies, however, mostly consist of small pilot-design studies [13-16]. As home monitoring is a relatively new modality in health care, most research is explorative, and randomized controlled trials and systematic reviews are scarce. An early systematic review of the literature, however, added value to the bariatric pathway, even though it is too early to draw final conclusions [17]. The willingness to participate in our study was high; this can be attributed to the small-scale design and, consequently, the close follow-up of the research team. Health technologies have the potential to provide additional support to a patient’s social system. However, many questions are yet to be answered. Results from this study indicate opportunities for future research. As described before, compliance might be an important factor in whether telehealth solutions in home environments or social systems will be effective. Future larger studies must explore whether patients are also willing to use telemonitoring with less involvement from a research team. Furthermore, it is still unclear which type of telehealth intervention patients prefer to use. In this study, physical, dietary, and psychosocial support was delivered all together. It is not clear which of these support types is most effectively facilitated by telehealth technologies. Not only the type but also the means of delivery of the intervention might be important. This varies based on the individuals. For instance, some might prefer a more direct approach (eg, automated push notifications for recipes around dinnertime), while others benefit more from an “on-demand” approach (eg, actively asking the chatbot for recipes). Future studies are recommended to determine the optimal type of support and means of delivery, or an optimal combination of both.

### Limitations

One of the drawbacks of this explorative approach was the number of participants, as well as the nonreproducibility of the results, as they were case-specific. Furthermore, an intelligent system was built with the full approval of the participants in this study, which could be limited by privacy concerns if applied on the general postbariatric population. Another limitation could be that the data found by trackers can be used by household members to check on each other instead of giving support. The substantial amount of data collected limited the analysis strategies. It remains challenging to process these data into a dashboard for each member of the coresponsibilities as well as distracting the focus of the obesity team.

### Conclusions

The results of this pilot study indicate that using data trackers in the home environment of patients could help the obesity team members to be better informed in their medical decision-making and, thus, lead to personalized support. On the other hand, there remains much room for wrong interpretations of the data. Nevertheless, this study made the first steps in an explorative way, leading to a modest conclusion.

## Appendix

Multimedia Appendix 1 Script of semistructured interviews.

## Abbreviations

MEC-U: Medical Research Ethics Committees United
UI: user interface

## References

[1] (2021). Obesity and overweight. World Health Organization.

[2] Sjöström L (2013). Review of the key results from the Swedish Obese Subjects (SOS) trial - a prospective controlled intervention study of bariatric surgery. J Intern Med.

[3] Ramos A, Kow L, Brown W, Welbourn R, Dixon J, Kinsman R, Waltonth IGRR (2019). 5th IFSO Global Registry Report.

[4] Luca P, Nicolas C, Marina V, Sarah B, Andrea L (2021). Where are my patients? Lost and found in bariatric surgery. Obes Surg.

[5] Lujan J, Tuero C, Landecho MF, Moncada R, A Cienfuegos J, Rotellar F, Silva C, Lapuente F, Martínez P, Frühbeck G, Valenti V (2020). Impact of routine and long-term follow-up on weight loss after bariatric surgery. Obes Surg.

[6] Vidal P, Ramón JM, Goday A, Parri A, Crous X, Trillo L, Pera M, Grande L (2014). Lack of adherence to follow-up visits after bariatric surgery: reasons and outcome. Obes Surg.

[7] Coulman KD, MacKichan F, Blazeby JM, Donovan JL, Owen-Smith A (2020). Patients' experiences of life after bariatric surgery and follow-up care: a qualitative study. BMJ Open.

[8] Neutelings I, Levy P, Djajadiningrat T, Hummels C (2017). Enhancing co-responsibility for patient engagement. Des J.

[9] Jansen JM, Niemantsverdriet K, Burghoorn AW, Lovei P, Neutelings I, Deckers E, Nienhuijs S (2020). Design for co-responsibility: connecting patients, partners, and professionals in bariatric lifestyle changes. Proceedings of the 2020 ACM Designing Interactive Systems Conference.

[10] Nienhuijs SW, Luijten AA, van Bavel H (2020). Transferring postbariatric patients to primary care: a regionwide analysis. Bariatr Surg Pract Patient Care.

[11] Bogers S, van Kollenburg KJ, Deckers E, Frens J, Hummels C (2018). A situated exploration of designing for personal health ecosystems through data-enabled design. Proceedings of the 2018 Designing Interactive Systems Conference.

[12] Motz V, Faust A, Dahmus J, Stern B, Soriano C, Stine JG (2021). utilization of a directly supervised telehealth-based exercise training program in patients with nonalcoholic steatohepatitis: feasibility study. JMIR Form Res.

[13] Nijland LMG, van Veen RN, Ruys AT, van Veldhuisen CL, Geerdink TH, de Castro SMM (2020). Feasibility of postoperative home monitoring using video consultation and vital sign monitoring of bariatric patients. Obes Surg.

[14] Bragg DD, Edis H, Clark S, Parsons SL, Perumpalath B, Lobo DN, Maxwell-Armstrong CA (2017). Development of a telehealth monitoring service after colorectal surgery: a feasibility study. World J Gastrointest Surg.

[15] Bradley LE, Forman EM, Kerrigan SG, Goldstein SP, Butryn ML, Thomas JG, Herbert JD, Sarwer DB (2017). project help: a remotely delivered behavioral intervention for weight regain after bariatric surgery. Obes Surg.

[16] Tenhagen M, van Ramshorst GH, Demirkiran A, Hunfeld MAJM, Cense HA (2016). Perioperative online weight monitoring in bariatric surgery with a digital internet-connected scale. Obes Surg.

[17] Coldebella B, Armfield NR, Bambling M, Hansen J, Edirippulige S (2018). The use of telemedicine for delivering healthcare to bariatric surgery patients: a literature review. J Telemed Telecare.

