# Supplementary material for: The Value of Tracking Data on the Behavior of Patients Who Have Undergone Bariatric Surgery: Explorative Study
Source: JMIR Form Res. 2022 May 6;6(5):e27389. doi: 10.2196/27389 (PMC9123534; doi:10.2196/27389)
Supplement: Multimedia Appendix 1 [file formative_v6i5e27389_app1.docx]

**Multimedia Appendix 1**

**Supplemental Material: Script for semi-structured interviews**

Note: translated for original Dutch script.

**Interviews**

Below are a few sample questions to give you an idea of what kind of questions are discussed in the interviews. The interviews have a semi-structured design and are based, among other things, on the gathered data point. The exact content therefore depends on a number of, yet unknown, topics. The interview also depends (in terms of content) on the specific situation of the patient and the partner. We will make a plan for this as soon as we have been able to view their bariatric data (whether or not the preliminary stage, assessment, pre- or post-operative, etc.) Every interview starts with an evaluation of how the research is going. Subsequently, based on the 'events' that week, it will be discussed what data has been received and what the participants' experiences are.

**Introduction conversation**

The first interview takes place at the hospital. The potential participants receive a general explanation about the project. This is an explanation of the purpose of the study, what is expected of the participants and the expected invasiveness. Potential participants who indicate that they are interested in participating will receive the information letter at home. The patients are indicated that they can always call or email if there are any further questions about the study.

**Phone call**

People who have received an information letter will be called after approximately 7 days. In this telephone conversation they are asked if they have understood all the information and if they have any additional questions. Then they are asked whether they want to confirm their participation or not. If they give a telephone confirmation, a concrete appointment is made for the first visit, in which we will go through and sign the information letter and the consent form together.

**WEEK 1:**

**General:**

- Explanation of study and practical matters.
- Go through the information letter and letter of consent.

Explanation about the study and the products of the first 2 weeks:

- Which products are installed.
- Which assignments the participants receive.
- What is expected of them.
- How to deal with uncertainties during the study.
- Explanation of how to use the App.
- Communication via the App and via telephone/mail.
- Agree when they will not be disturbed by questions via the app.
- Handing over study information (instructions for use, privacy statements) and explanations.

**Contents of interview:**

*History:*

Goal: get to know the patient and partner better. Go deeper into bariatric history by asking questions such as:

- Can you tell us about your history of obesity?
- Have you always been a bit heavier, or did it start at some point?
- How old were you and what stage of your life were you? Reference to Rand36 and screening questionnaire.
- Does obesity occurs in your family?
- Can you tell us how you came to choose gastric bypass surgery?
- Do others know that you will have surgery soon?
- What did you do with the information you received from the hospital?
- Are you working with ModiFast?
- Is it going well or is it difficult? Can you explain why?
- How is your family doing now that you need to make changes? What does your family think?
- You had a stomach reduction operation a while ago, how long ago was that?
- How are you now?
- What were your expectations for your life after gastric bypass surgery? And do these correspond to how it is now?
- In what positive ways has your life changed?
- Are there things that you don't like because of the surgery? Things you would like to do, but are now unable to do or can no longer do because of the surgery?
- We understand that people undergoing bariatric surgery have to change their lifestyle in many ways, has that been successful for you?
- What went easy/what did you struggle with? And how is that going now?
- Are there people in your social environment who have supported you in this process? How did they do this?
- Turn to partner: how are you?
- What was it like for you that your partner had gastric bypass surgery?
- Have many things changed for you?
- Are there times when it has been difficult for you?
- What positive things has it brought you?
- Has the change also had an effect on your own lifestyle?

*Current status*

Present and daily routines. Mapping the daily routines of the family using cultural probes.

- What does a 'normal' week look like for you?
- Do you work?
- What hobbies do you have?
- What do you do in your spare time?
- When do you eat (together/with other people)?
- What time do you usually go to sleep?
- When will you get up again?
- Where do you usually eat?
- What are pleasant moments during the day?
- What are difficult moments?
- How is your lifestyle and lifestyle-change (in relation to the time of the treatment phase)?
- What positively influences a normal day (in relation to your illness)?
- What negatively affects a normal day (in relation to your illness)?

*Future:*

- What do you expect from participating in this study?
- For people who are in the preoperative trajectory
- What are your expectations for your life after surgery?
- What do you dread the most?
- What do you think will go well?
- Do you have people in your social environment who can support you in this? Who are they? And how do you expect them to support you?
- Turning to partner
- What do you think about your partner having gastric bypass surgery?
- What are your expectations for the future?
- What do you think will change for you?
- For people who are in the postoperative process
- What are your expectations for the future?
- What do you dread the most?
- What do you think will go well?

*Closing the interview*

- Do you have any questions for us? Possible practical matters for the coming weeks:
- Make an appointment for the next visit
- Thank you very much for your time. We'll see you again in two weeks.

**WEEK 2**

**Last week's evaluation:**

- How are you?
- How did you like the first week of the study – in general?
- Are all assignments clear and have they been carried out successfully? If not, can you explain what went wrong?
- Do all products work?
- Is it possible to charge the products properly?
- Specific per product: how do you like using this product? Interesting/boring/simple/difficult/fun/dislike/etc.
- What do you think of the questions you get?
- How did you like the 'amount of work/time lost with the study'?
- In the past week, have you felt that you were disturbed by the app?
- You have indicated these moments as moments when you do not want to be disturbed: […]. Is this still correct, or do you want to change something?

**Getting to know the routines of the participants – moving on if it wasn't completed last week.**

Discussing the data we have collected through the products:

- Last week we received data from the products we gave you and we now want to discuss it with you.
- [example] The accelerometer on the refrigerator shows that there are certain times when the refrigerator opens and closes a lot, we also see that […]. Can you explain what we see there?
- [example] We have noticed that the television often comes on at night. Can you tell us more about that?
- [example] In the App you indicated that you have difficulty with […], can you explain why this is?
- [example] We see that your partner indicates that you have trouble sticking to your diet when the children sit down to eat sweets. Do you see it that way and can you tell us more about it?

**Closing the interview:**

- What questions do you have for us?
- Give patient and partner the opportunity to discuss any ambiguities.

**Possible practical matters for the coming weeks:**

- You have indicated that you find the use of the product […] annoying. We will take these again.
- We brought a new product that you can use in the coming weeks. It is about product […]. [explanation and try out new product]
- Repeat appointments for the coming weeks: As we have just agreed, we will no longer disturb you at the following moments: […]. In addition, we have given you this new product […]. If you have any questions about the use of this in the meantime, please contact us via […].
- Make an appointment for the next visit
- Thank you very much for your time. We'll see you again in two weeks.

**WEEK 4**

**Practical issues:**

Last week's evaluation:

- How are you?
- How did you like the past weeks of the study – in general?
- Are all assignments clear and have they been carried out successfully? If not, can you explain what went wrong?
- Do all products work?
- Is it possible to charge the products properly?
- Specific per product: how do you like using this product? Interesting/boring/simple/difficult/fun/dislike/etc.
- What do you think of the questions you get?
- How did you like the 'amount of work/time lost with the study'?
- In the past week, have you felt that you were disturbed by the app?
- You have indicated these moments as moments when you do not want to be disturbed: […]. Is this still correct, or do you want to change something?

**Discussing the data points:**

- [example] We have noticed that you have started to move more and more, can you tell us why?
- [example] We saw that your partner (the patient) indicated that it helps if you go shopping with them. Can you explain why?
- [example] In the app you indicated that you have difficulty with […], can you explain why?
- [example] Which day was a really good day for you, and why?
- [example] And which day or which moment from the past week was less? Why?

**Closing the interview**

- What questions do you have for us?
- Give the patient and partner the opportunity to discuss any uncertainties.

**Possible practical matters for the coming weeks:**

- You have indicated that you find the use of the product […] annoying. We will take these again.
- Last time we were here you indicated that you think that […] can also be relevant to measure. We've now brought you an extra accelerometer so we can try it out.
- We brought a new product that you can use in the coming weeks. It is about product […]. [explanation and try out new product]
- Repeat appointments for the coming weeks: As we have just agreed, we will no longer disturb you at the following moments: […]. In addition, we have given you this new product […]. If you have any questions about the use of this in the meantime, please contact us via […].
- Make an appointment for the next visit
- Thank you very much for your time. We'll see you again in two weeks.

**WEEK 6**

**Last week's evaluation:**

- How are you?
- How has it been in the last few weeks?
- Are all assignments clear and have they been carried out successfully? If not, can you explain what went wrong?
- Do all products work?
- Specific per product: how did you like using this product? Interesting/boring/simple/difficult/fun/dislike/etc.
- What do you think of the questions you have received?
- How did you like the 'amount of work/time lost with the study'?
- In the past week, have you felt that you were disturbed by the app?

**Discussing the data points:**

- [example] We see in the data that there was a lot of activity in the kitchen on Wednesday. Can you tell us what happened that night? Who cooked? Who ate with them? What was cooked?
- [example] In the app we asked you questions about the stomach and head hunger. In the case of stomach hunger, we also saw that you indicated in the App that you found it difficult to deal with it. Can you tell us something about that?

**Evaluation over the entire participation period**

- Can you give a general impression of your experiences with the products throughout the study?
- Can you give some examples of positive experiences you have had in the last 6 weeks?
- Can you give some examples of things that bothered you in the last 6 weeks?
- In the first week you mentioned that you expected [...] from participating in the study, does this correspond somewhat with your experiences?
- Did you learn new things about yourself while participating in this study?
- After participating in this study, if you could get all the information/data you could want, what information do you think would really help you better stick to your new lifestyle for the rest of your life?
- And if you could get all the products you want, what products could help you maintain your new lifestyle for years to come?
- Could your social environment help you with this even better, and in what way?

**Closing the interview:**

- Give the patient and partner the opportunity to discuss any uncertainties.

**End of study:**

- Thank you very much for your time and all the information you have shared with us. We will further analyze and process this information.
- You will receive a booklet from us containing an overview of the information we have collected together with you. You will receive this […]
- [example] We previously took this picture of your kitchen. We would like to use this photo for publications. Are you okay with that? If so, would you please sign this additional consent form? [Go through and sign the consent form together.]
- We take retrieve all products
